# Gene signatures of cyclin-dependent kinases: a comparative study in naïve early and advanced stages of lung metastasis breast cancer among pre- and post-menopausal women

**DOI:** 10.18632/genesandcancer.209

**Published:** 2021-02-10

**Authors:** Muhammad Fazal Hussain Qureshi, Muzna Shah, Mahira Lakhani, Zain Jawed Abubaker, Danish Mohammad, Hira Farhan, Iman Zia, Rida Tafveez, Samahir Tariq Khan, Ghani Rubina, Mushtaq Shamim, Haider Ghulam

**Affiliations:** ^1^ Medical Students, Ziauddin University, Clifton, Karachi, Pakistan; ^2^ Department of Biochemistry, Sohail University, Karachi, Pakistan; ^3^ Department of Biochemistry, Ziauddin University, Clifton, Karachi, Pakistan; ^4^ Oncology Department, Jinnah Postgraduate Medical Center, Karachi, Pakistan

**Keywords:** cyclin-dependent kinases, breast cancer, menopause, human epidermal growth factor receptor 2

## Abstract

The Human epidermal growth factor receptor 2 positive (HER2+) breast cancer (BC) is a more aggressive tumor with 5 years median survival rates after metastasis. Despite successful treatment, unfortunately, the majority of affected patients die. Defects in cell cycle and transcription regulation phases which are governed by cyclin-dependent kinases (CDKs) are the hallmark of many cancers that underpinning the progression of the disease. Therefore, the current study looked at the alteration of six CDKs mRNA expression levels in pre- and postmenopausal lung metastasis BC groups; the majority were HER2+. Two hundred pre-and postmenopausal lung metastasis breast cancer and healthy control blood samples were taken for RNA isolation. Quantitative PCR was done for CDKs mRNA expressions. We observed overexpression of CDK11, CDK12, CDK17, CDK18, and CDK19 in both pre- and postmenopausal groups. However, CDK20 showed progressive downregulation from early to advanced stages in both groups of patients. Collectively, this data revealed that CDKs overexpression levels may predict BC disease progression and provide further rationale for novel anticancer strategies for HER2+ BC cancers.

## INTRODUCTION

Breast cancer (BC) in women is a severe health burden worldwide and responsible for about 375,000 deaths in the year 2000. It is a major cause of cancer in both high- and low-income countries [1]. Many studies have shown poor prognosis at a young age. Cancer in this age group is more aggressive [2-4]. In Asia and other lesser developed countries, there is a lack of adequate services and treatment for BC, hence a higher mortality rate is observed when compared to a developed setup [5-7].

In Pakistan, Karachi is supposed to have the highest incidence of BC among the Asian population [8]. Nonetheless, the age-standardized incidence rate of BC is highest in Pakistan among Asian countries [9]. The BC lung metastatic relapse has become a serious clinical problem due to a lack of treatment, shorter survival time, and a high mortality rate [10-13]. Unfortunately, due to late diagnosis, the mortality rate in BC is high in Pakistan. Currently, there is no data available regarding the most occurring metastatic site in BC Pakistani patients. Human epidermal growth factor receptor 2 (HER2) known as an oncogene, confers a more aggressive tumor and is associated with an increased rate of recurrence, mortality, and metastatic disease despite the remarkable progress in treatment [14-15]. Unfortunately, at advanced stages, patients might fail to respond to targeted therapies. Patients may also have an initial response followed by development of resistance. Therefore, a strategy that investigates the target transcription elements might be more effective in HER2 positive BC patients. Moreover, there is no progress in BC in recent times. To generate accurate data, there is an urgent requirement to discover diagnostic, prognostic non-invasive blood biomarkers for predicting clinical outcomes at an early stage and selecting patients who could benefit after treatment. It has been postulated that various alternative pathways like cell-cycle dysregulation, apoptosis, growth factor and androgen receptor pathways are involved in breast cancer [16-21].

Cyclin-dependent kinases (CDKs) regulate the cell cycle progression and transcriptional regulation which is frequently dysregulated in all human malignancies. They are an attractive target for BC therapeutic strategy [22-23]. Therefore, they have been the interest of the current study. More recently, a CDK4/6 inhibitor, palbociclib was approved by the FDA for treating metastatic breast cancer [24-25]. Overexpression, dysregulation, and mutation of these CDKs contribute to the proliferation of cancer cells and inhibition can lead to both cell cycle arrest and apoptosis [26].

CDK11 (formerly named PITSLRE) is an essential CDK for cell survival, and its increased expression in various cancers is associated with poor prognosis [27-29]. While CDK12 is known to protect the normal cell from DNA damage and is involved in the regulation of RNA polymerase II (RNApol II) [30-31]. In contrast to other cell cycle CDKs family regulators, CDK11, CDK12, CDK19, and CDK20 are the transcriptional regulators that control fundamental cellular processes. Furthermore, previously CDK12 functional mutation was reported which hampered its ability to promote tumor suppressor gene in ovarian carcinoma [32]. Recent data shows the highest expression of CDK19 in metastatic prostate cancer. However, a past study revealed novel links between CDK19 and cell proliferation through repression of p53 response [33-34]. CDK20 is a newly identified human CDK family protein that is known to control cell cycle progression in various cancers [35-36].

There is little progress on the CDK17 and CDK18 in recent times. Their role in the cell cycle and expression in BC remains unclear. Moreover, the role of CDK11, CDK12, CDK19, and CDK20 in metastatic BC and molecular mechanisms associated with CDKs mRNA expression are poorly understood. The advanced stage of BC is a crucial clinical problem and poses a challenge to the clinician. Thus, the present study aims to investigate the six CDKs 11, 12, 17, 18, 19 and 20 mRNA expression at an early stage (II) and advanced stage (III and IV) with lung metastasis patients; early detection for new approaches to treat pre- and postmenopausal naïve BC lung metastatic.

## RESULTS

Patients were divided into two groups based on their menopausal status as pre-menopausal and postmenopausal associated with lung metastasis. We quantified six CDKs gene transcripts in both groups at early (II) and advanced stages (III & IV). The mean age for pre-menopausal participants was 30 with a standard deviation of 3.8 years, while the mean age of postmenopausal participants was 45, with a standard deviation of 2.8 years.

90% of the participants reported no prior family history of breast cancer. Out of 150 pre-menopausal BC patients, 140 were HER-2+, and 10 were negative while among 100 postmenopausal 90 were HER-2+, and 05 were negative. Detailed clinicopathological characteristics of the studied cases are presented in Table 1.

**Figure 1:**
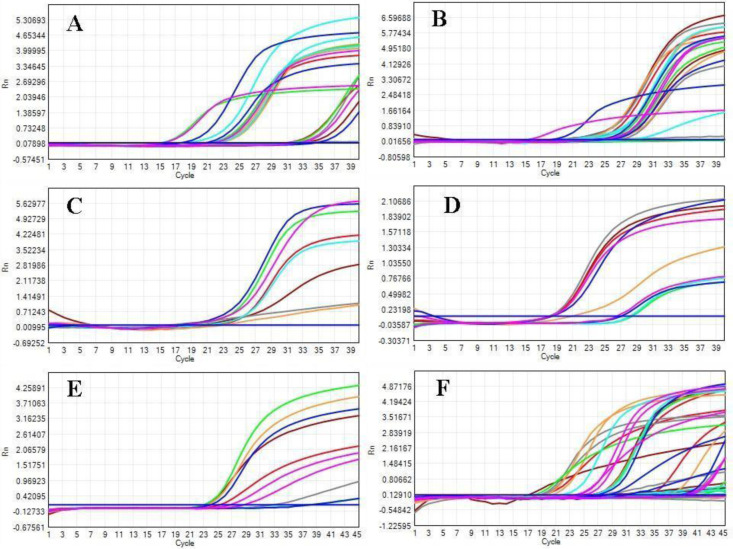
An amplification curves of CDKs showing respective Ct values for two distinctive stages early (II) and advanced stages (II & IV) of pre-and postmenopausal BC patients. A. CDK 11 B. CDK 12, C. CDK17, D. CDK18, E. CDK F. CDK 20.

**Figure 2:**
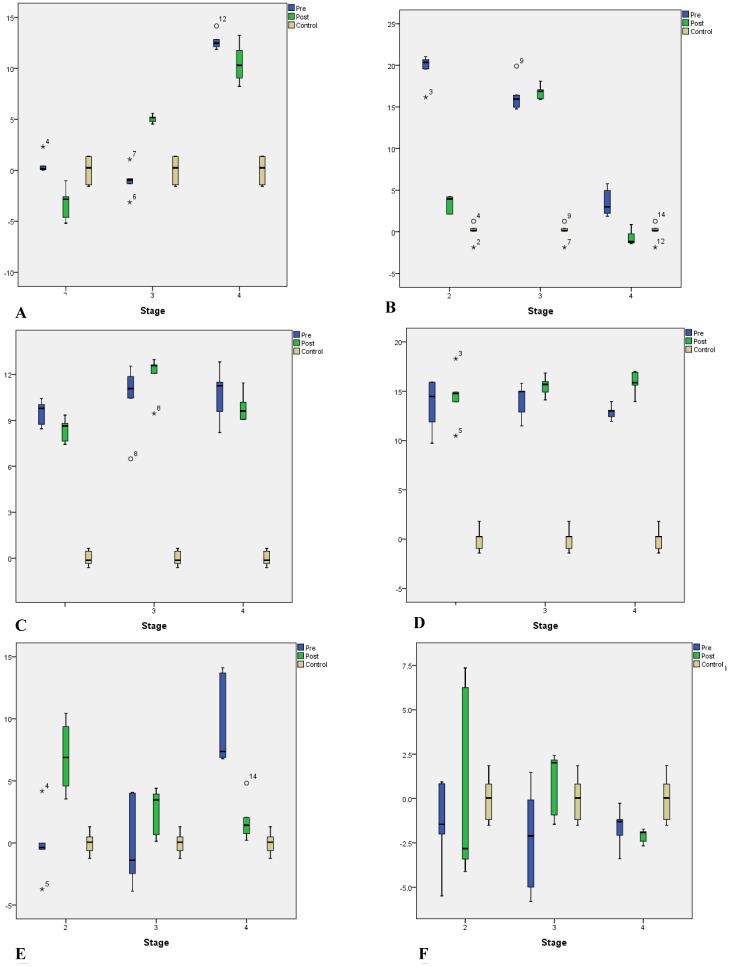
Box plots showing CDKs relative expressions ▲▲Ct values of control and all pre- and postmenopausal BC groups at early and advanced stages validated by qPCR. All data were normalized against levels of GAPDH mRNA expression within the BC and control samples. Error bars indicate ± SD, P < 0.005 after three individual experiments. A. The CDK 11, B. CDK 12, C. CDK17, D. CDK 18, E. CDK 19, and F. CDK 20.

The total RNA was isolated from each pre- and postmenopausal BC blood samples and primers used for this study are summarised in table 2. To investigate the role of CDKs in different stages of BC, the mRNA expression of CDKs [11, 12, 17, 18, 19, 20] was measured using RT-qPCR. The cycle threshold (Ct) values (Figure 1) were used to determine the amount of mRNA and ▲Ct values were then determined by calculating the median absolute deviation for all the samples within the same experimental group. Samples that did not correlate with the results were declared as outliers and excluded from the analysis. Relative change for expression of marker and housekeeping gene (GAPDH) was determined by the Levene equation (▲▲Ct values). ANOVA was used to determine the statistical significance of the observed fold change, and a p-value of less than 0.05 was considered as significant. The relative fold change in the CDKs mRNA expression level of pre- and postmenopausal BC was shown as the 2^-▲▲Ct values. The average gene expression values (▲Ct) were compared between the healthy control group and the BC patients (Figure 2). ANOVA and Post-hoc Tukey test was applied to determine intergroup group means and variations.

In the pre-menopausal BC group (Tables 3 & 4), the relative gene expression of CDK11 (Figure 2A) was found to be high in advanced stages (IV) only, with statistical significance (*p* = 0.0001). However, in the postmenopausal group, it showed progressively increased expression from stage III to stage IV with statistical significance (*p* = 0.001).

We found CDK12 up-regulated at both early and advanced stages in the pre-menopausal group. Early-stage did not reach statistical significance (*p* = 0.06). Interestingly, in the postmenopausal group, upregulation was observed in CDK12 only in advanced stages, and stage IV did not show statistical significance (*p* = 0.06).

**Table 1:** Table 1: Study set and clinicopathological features

Variables	Pre-menopausal BC	Postmenopausal BC
No of BC patients	150	100
Mean age at sample draw (years; range)	30 ± 3.8	45 ± 2.8
Tumour grade (number of cases) 1 2 3	55 35 60	35 35 30
Node status (number of cases) N0 N1	70 25	80 20
Stage (number of cases) II III IV	50 50 50	35 35 30
HER-2 status Positive Negative	140 10	90 05
Metastasis (number of cases) M0 M1	80 50	60 30

**Table 2:** Table 2: Primers sequences used in qPCR

Target gene	Primer Sequences
CDK11	F’ AGAACATATTCGACTCTCCAGCACT R’ GAGTATTCCTTAGCACCAAGCAGTA
CDK12	F’ TGG ACT TGC TCG GCT CTA TAA CTC R’ CCC AAG AAT ACA TCC ACA GCT CCA
CDK17	F’ CATAGACGGATCTCAATGGAGGA F’ TGGTTGGTCAAATGGTGGACT
CDK18	F’ GAGTTCCGCACCTACAGCTTCC R’GCCTCGGTAGCCTGGGTCCTT
CDK19	F’ CGGAACCTATTTTTCACTGTCG R’ TGTGGGATATTCTGGCATCTT
CDK20	F’ TCCAAGGCTCTCCTCCATCA R’ TCTGGGTTCAACAGCGACTC

The mRNA expression level of CDK17 in the pre-menopausal group was overexpressed with a gradual increase in expression in advanced stages compared to the early stage. We also found a statistically significant increase (*p* = 0.001) in mRNA expression of postmenopausal patients at an advanced stage (III) compared to stage IV; the early stage did not reach statistical significance (*p* = 0.098).

The statistically significant approach was seen in CDK18 mRNA expression, which was up-regulated in all stages among pre- and postmenopausal groups.

CDK19 was overexpressed only in stage IV of the pre-menopausal group with *p* = 0.002. The postmenopausal group showed a 5.52-fold increase of CDK19 expression at an advanced stage (IV) only, with statistical significance *p* = 0.04. Whereas CDK20 showed downregulation progressively from early to advanced stages in pre- and postmenopausal.

## DISCUSSION

Our current study has revealed six CDKs gene signatures in BC among pre and postmenopausal women at early (II) and advanced stages (III & IV) with lung metastasis. Among 250 BC patients, we found 150 pre-menopausal (mean age 30 ± 3.8) and rest were postmenopausal (mean age 45 ± 2.8). According to a survey conducted in Karachi, the average incidence of BC presentation was 27.4% between the age of 25-37 years, which was highest compared to other cancer presentation age groups [8]. As to why the rates of pre-menopausal breast cancer in Pakistan are so high is not yet known. It can occur due to delay in seeking medical attention, a conservative society, and religious beliefs that do not permit talks about BC in our setup.

The principal regulators of various cellular processes, CDKs personify the cell molecular machinery, associated with both cell cycle and transcription phases which represent typical oncogenes and tumor suppressors. However, deranges reflect an unscheduled division of the cell, which could lead to uncontrolled proliferation and tumorigenesis. To the best of our knowledge, this is the first study showing six CDKs mRNA expressions in pre- and postmenopausal BC patients associated with lung metastasis.

It is recognized that CDK18/ PCTK3 is required for S phase transit, and its inhibition leads to DNA damage, defects in replication, and chromosomal abnormalities [37]. The most significant finding of this study is the overexpression of CDK18 found at both early and advanced stages in two groups. However, in the postmenopausal group, it exhibited a slight up-regulation in advanced stages. It is difficult to disseminate if these changes are actively involved during tumorigenesis or it occurs due to genetic alteration. A study showed that anti-HER2 therapies improved the outcome of metastatic BC [38], while metastatic HER2+ is still an incurable disease as patients showed resistance to current treatment that could lead to tumor progression and death [39]. In support of our findings, a recent study also observed higher levels of CDK18 mRNA and protein expression in BC compared to the normal breast [40].

CDK 17, also known as PCTAIRE-2/PCTK2, was not studied much in cancer. However, recently it was considered a potential inhibitor of autophagy implicated in several pathological conditions such as cancer, neurodegenerative diseases, and infections [41]. Moreover, its increased expression was also found in Alzheimer’s disease, promoting neurodegeneration [42]. We also observed CDK17 overexpression in advanced stages of pre- and postmenopausal BC group. Therefore, in support of previously reported studies, we can predict that CDK17 upregulation may be required to stimulate autophagy, an event frequently found in breast tumorigenesis [41].

**Table 3:** Table 3: ▲Ct mean, ▲▲Ct, 2−▲▲Ct and p values for CDKs differentially expressed gene in early and advanced stage BC Pre-menopausal groups

**CDKs**	**Early-stage**					**Advanced stages**									
	**II**					**III**					**IV**				
	**▲Ct mean BC**	**▲Ct mean control**	**▲▲ Ct**	**2^- ▲▲Ct**	**p-Value**	**▲Ct mean BC**	**▲Ct mean control**	**▲▲ Ct**	**2^-▲▲Ct**	***p*-Value**	**▲Ct mean BC**	**▲Ct mean control**	**▲▲ Ct**	**2^-▲▲Ct**	***p*-Value**
11	2.19	1.56	0.63	0.646	0.04	2.59	1.56	1.03	0.489	0.001	14.29	1.56	12.73	1.47×10^-4^	0.0001
12	17.40	2.21	15.19	2.67×10^-5^	0.067	15.21	3.41	11.80	2.80×10^-4^	0.001	18.25	2.60	15.65	1.94×10^-5^	0.0001
17	13.81	4.32	9.49	1.3×10^-3^	0.001	14.81	4.32	10.49	6.95×10^-4^	0.0001	16.63	3.75	12.88	1.32×10^-4^	0.001
18	19.21	1.11	18.1	3.5×10^-6^	0.04	18.90	1.41	17.4	5.7×10^-6^	0.04	16.63	3.75	12.88	1.32×10^-4^	0.0001
19	6.28	1.48	4.8	0.035	0.02	4.45	0.95	3.5	0.088	0.001	14.15	3.65	10.5	6.90×10^-4^	0.002
20	1.41	0.47	0.94	0.521	0.05	1.50	0.89	0.61	0.655	0.05	1.20	0.23	0.97	0.510	0.04

*p < 0.05 = statistically significant; Ct = Threshold cycle

**Table 4:** Table 4: ▲Ct mean, ▲▲Ct, 2−▲▲Ct and p values for CDKs differentially expressed gene in early and advanced stage BC Post-menopausal groups

**CDKs**	**Early-stage**					**Advanced stages**									
	**II**					**III**					**IV**				
	**▲Ct mean BC**	**▲Ct mean control**	**▲▲ Ct**	**2^- ▲▲Ct**	**p-Value**	**▲Ct mean BC**	**▲Ct mean control**	**▲▲ Ct**	**2^-▲▲Ct**	***p*-Value**	**▲Ct mean BC**	**▲Ct mean control**	**▲▲ Ct**	**2^-▲▲Ct**	***p*-Value**
11	1.65	1.56	0.09	0.939	0.001	6.66	1.59	5.06	0.029	0.001	12.12	1.56	10.52	6.8×10^-4^	0.001
12	4.28	3.33	0.95	0.517	0.047	14.57	2.21	12.41	1.83×10^-4^	0.01	11.87	2.21	9.66	1.23×10^-3^	0.06
17	12.69	4.32	8.37	3.0×10^-3^	0.098	16.25	4.32	11.93	2.56×10^-4^	0.04	15.19	4.32	10.87	5.34×10^-4^	0.001
18	18.22	0.32	17.91	4.0×10^-6^	0.03	19.26	0.96	18.31	3.0×10^-6^	0.014	19.62	0.71	18.91	2.04×10^-6^	0.001
19	14.34	2.81	11.50	3.45×10^-4^	0.001	15.12	5.32	9.8	1.12×10^-3^	0.001	16.23	8.73	7.51	5.52	0.04
20	2.06	1.41	0.65	0.637	0.02	4.52	1.01	3.50	0.088	0.06	1.41	0.70	0.71	0.611	0.04

*p < 0.05 = statistically significant; Ct = Threshold cycle.

The CDK19, homolog CDK8, contributes to the first stage of gene expression by forming a mediator complex that links to transcription factor Pol II to regulate different transcription programs [43-44]. However, a study identified novel links between CDK19, cell proliferation, and p53 response [34]. In the current study, CDK19 upregulation is noted in the pre-menopausal group compared to the postmenopausal BC group. Furthermore, a previous study has also focused on high expression of both CDK8 and CDK19 in prostate cancer metastases [45].

CDK20 also known as CCRK, which has sequence homology to CDK7, represents a novel cell cycle-related kinase (CAK) activity, and controls cell cycle progression [46] in various cancers including lung [47], colorectal, liver and ovarian cancer [36, 48-50]. More recently, Bowen Sun et al. revealed that CDK7 inhibition blocks the activation of genes associated with HER2 inhibitor-resistant (HER2iR) in BC xenograft models in vivo [51]. Moreover, further study revealed that CDK20 results in EZH2 (zeste homolog 2) upregulation and may be associated with poor survival in liver cancer [49]. Precisely how CDK20 functions in breast cancer remain largely unknown. Consistent with these studies, we tried to explore the function of CDK20 in BC early and advanced stages. It was found that it was highly expressed only in advanced stage (IV) of the pre-menopausal group compared to postmenopausal BC group. The Kelch-like ECH-associated protein 1 (KEAP1)–nuclear factor erythroid-2-related factor 2 (NRF2) cytoprotective pathway regulates cell growth, cell metabolism, cell proliferation, elimination of reactive oxygen species (ROS), gene transcription, essential events in cellular redox homeostasis and tumorigenesis. CDK20 modulates the KEAP1–NRF2 cytoprotective pathway and regulates tumor progression, implying that it is a potent therapeutic target for lung cancer [47]. This would suggest that overexpression of CDK20 at an advanced stage of lung metastasis in pre-menopausal BC may regulate KEAP1–NRF2 cytoprotective pathway. On this account, it will be interesting to investigate the expression in different treated BC patients in the future.

We also observed overexpression of CDK11 at an advanced stage (IV) in both pre-and postmenopausal BC groups compared to the early stage. The CDK11 is involved in transcription and RNA processing events by interacting with its regulatory partner cyclin L [52-54], but also is implicated in other cellular processes, including autophagy [53]. Similar to our results, Zhou Y and his group found elevated CDK11p110 expression in breast cancer tissues significantly correlating with poor differentiation and was also associated with clinicopathological characteristics of breast cancer patients [54]. Our results suggest that elevated CDK11 expression might be crucial for the proliferation and growth of human breast cancer metastasis.

The CDK12 (CRKRS, CRKR, or CRK7) is ubiquitously expressed, in different human tissues [56]; higher expression was seen in male and female endocrine tissue, reproductive tissue, spleen and bone marrow mainly localized in the nucleus [57]. Inactivation of the CDK12 gene has been associated with a unique genomic instability pattern that could lead to tumorigenesis [59]. Furthermore, CDK12 also lethally interacts with proto-oncogenes such as MYC and EWS/ FLI (Ewing sarcoma (ES) oncoprotein) and these interactions also provide an opportunity for a therapeutic target [59-60]. The current study showed a high expression of CDK12 at early and advanced stages of pre- and postmenopausal group, but only slightly increased in the pre-menopausal group. The question remains unclear whether these alterations are due to the mutation or interaction with some oncogenes.

Taken together, clinical studies on CDKs inhibitors, are needed to investigate the role of these CDKs in advanced stages HER2+ BC patients. Furthermore, clinical trials will also clarify whether these CDKs will become targets for the treatment of metastatic HER2+BC patients.

## MATERIALS AND METHODS

### Ethical statement

Breast cancer (BC) blood samples were obtained from Ziauddin Hospital and Jinnah Postgraduate Medical Centre (JPMC), Karachi. All the patients enrolled in the current study signed the consent form. The study was approved by the Ethics Review Committee (ERC) of Ziauddin University (Reference No: 0220518S MBC).

### Patient recruitments and sample collection

Peripheral blood (5ml) was collected from pre-menopausal (*n* = 100) ranging from 30 to 44 years of age, postmenopausal (*n* = 100) ranging from 45 to 60 years of age naïve (untreated) BC patients. The healthy controls (*n* = 50) with a similar distribution of age were recruited from the same hospital. The whole blood in EDTA tubes was stored at −20 °C in the freezer.

Postmenopausal status was confirmed, those who had no menstrual flow for 12 months, and rest considered as pre-menopausal. Patients characteristics are shown in Table 1. Briefly, naïve BC patients were selected based on the mammographic appearance of the tumor, basal phenotype, and the histologic tumor distribution together with the conventional tumor attributes (tumor size, axillary node status, and histologic malignancy grade). Using the TNM system, staging for each case will be determined, tumor size (T), spread to the lymph nodes (N), and whether the tumor metastasized (M).

Among them, patients with stage II were grouped as early-stage pre- and post-menopause BC. The advanced stage pre and post-menopause BC group comprised of patients with stages III and IV (lung metastasis); metastatic changes start during stage III leading it to stage IV.

Women who had undergone hysterectomy, had any ovarian problems, had a personal history of any other cancer, or had undetermined menopausal state were excluded from the study. Immunohistochemical staining was done on paraffin sections and stained with antibodies against ER; PR and HER2. Immunohistochemical results were scored, and tumors were considered positive for hormone receptors if at least 1% of the tumor cells showed nuclear staining. According to the guidelines for the assessment of HER2 status, staining for HER2 will be scored: 0, no staining; 1+, (weak staining in < 10% of tumor cells), 2+, moderately strong membrane staining in >10% of the tumor cells; 3+, strong membrane staining in >10% of the tumor cells.

### RNA Extraction and RT-qPCR analysis

Total RNA was extracted from whole blood of pre and postmenopausal naïve BC patients at early (II) and advanced stages (III & IV) using an extraction kit (VIOGENE, USA) according to the manufacturer’s protocol and immediately stored at -80 C. The isolated 5ng RNA was reverse transcribed into cDNA using innuSCRIPT One Step RT_PCR SyGreen kit (ANALYTIK JENA). Primers listed in Table 2.

Briefly, RT-qPCR was performed using 1µl of cDNA and 0.2 µM primers in 10µl of qPCR mixture. The PCR cycle conditions were as follows: preincubation for 10 seconds at 95 °C, annealing at 60°C for 30 seconds, and extension at 72°C for 1 minute followed by 40 cycles. Each sample was analyzed in triplicate and the mean of three experiments value used as the relative quantification.

The relative expression of all CDKs determined via ▲▲Ct method, which normalized with reference https://pubmed.ncbi.nlm.nih.gov/

(GAPDH). The threhold cycle (Ct) was manually calculated for each CDK gene (gene of interest) and normalized to GAPDH. Relative expression levels of each target CDK gene expressed as ▲Ct, which was calculated as ▲Ct = Ct gene of interest – Ct Reference gene (GAPDH). However relative expression calculated as ▲Ct (gene of interest)- ▲Ct (Reference gene) =▲▲Ct.

### Statistical analysis

For statistical analysis, the ANOVA test was used to analyze the significance of the differential expression pattern. The significance was defined as a p-value < 0.05. Shapiro-Wilk test used to analyze the difference in the expression level between two groups (pre-menopausal and postmenopausal) and ▲Ct values were obtained for each stage and control group.

## Abbreviations

CDK: cyclin-dependent kinases
BC: breast cancer
HER2+: Human epidermal growth factor receptor 2 positive
Ct: Cycle threshold
ER: Estrogen receptors
PR: progesterone receptor
GAPDH: Glyceraldehyde 3-phosphate dehydrogenase
TNM: Tumour, Node, Metastasis
